# Life‐Course Behavioral Changes: Impact on Cognitive Performance, Resilience, and Disease Biomarkers in Late‐Life

**DOI:** 10.1002/alz70860_103511

**Published:** 2025-12-23

**Authors:** Magdalena I Tolea, Mahesh S Joshi, James E Galvin

**Affiliations:** ^1^ University of Miami Miller School of Medicine, Boca Raton, FL, USA

## Abstract

**Background:**

Healthy lifestyles such as regular exercise, diet, social and cognitive activity may reduce dementia risk by helping build greater cognitive reserve across the life course, promoting resilience to neuropathology and better cognitive outcomes in late‐life. However, tracking these health behaviors throughout the life‐course (age 25, 40, 65) is difficult. We captured self‐reported health behaviors through the life‐course and evaluated their relationship with cognitive performance, resilience, and disease biomarkers in late‐life.

**Method:**

In 378 adults aged 50+yrs, we assessed the impact of change in physical activity (PA), cognitive activity (CA), social activity (SA), and diet from age 25 to present age on global cognitive performance (MoCA), resilience (Resilience Index), and biomarkers (plasma ptau217% ratio, NFL, GFAP; MR volumetric data). Behaviors were categorized as improved/no change vs declined and pairs of lifestyle behavior (e.g., physical activity and diet) were categorized as improved/no change in both, improved/no change in one, vs decline in both. Relationships between healthy behavioral change and MoCA, resilience, and biomarkers, and mediation of relationships between behavioral changes and MoCA were tested with age‐adjusted GLM models.

**Result:**

Self‐reported healthy change in diet from age 25 was positively associated with MoCA score (β=1.51±0.48;p=0.002), accounting for age differences and multiple comparisons. A synergistic effect was found when changes in diet were combined with healthy changes in PA (β=3.20±0.76;p<0.0001) and SA (β=2.74±0.71;p<0.0001). Healthy changes in PA (β=13.79±4.23;p=0.001) and SA (β=17.93±3.97; *p* <0.0001) were related to resilience and their effects were synergistically increased when healthy changes in diet were also reported. Beta coefficients were reduced by 15‐43% in resilience mediation models. Amygdala volume was positively associated with healthy changes in SA (β=0.13±0.05;p=0.007) with a trend for greater amygdala (β=0.10±0.05;p=0.054) and entorhinal area volume (β=0.24±0.14;p=0.088) in those reporting healthy change in PA.

**Conclusion:**

Findings suggest that improving/maintaining diet into late‐life, alone and especially in combination with improved physical and social activity level, increases resilience to neurodegenerative diseases and leads to better cognitive performance in later life. Evidence of greater amygdala volume in those reporting improved social and physical activity provides further support for the beneficial effect of maintaining lifelong positive lifestyle practices on brain health.

